# Relationships between vitamin C intake and COPD assessed by machine learning approaches from the NHANES (2017–2023)

**DOI:** 10.3389/fnut.2025.1563692

**Published:** 2025-05-15

**Authors:** Xinxin Tao, Xianwei Ye

**Affiliations:** ^1^School of Clinical Medicine, Guizhou Medical University, Guiyang, Guizhou, China; ^2^Department of Respiratory and Critical Care Medicine, Guizhou Provincial People’s Hospital, Guiyang, Guizhou, China

**Keywords:** chronic obstructive pulmonary disease, vitamin C intake, LASSO, Random Forest, XGBoost, RCS

## Abstract

**Background:**

This research aims to explore the possible link between Vitamin C Intake (VCI) and the incidence of Chronic Obstructive Pulmonary Disease (COPD) in Americans aged over 20.

**Methods:**

This study analyzed data from 10,757 participants with or without COPD from NHANES (2017–2023). The primary exposure variable, VCI, was grouped by quartiles. Missing data were handled via multiple imputations. A Directed Acyclic Graph (DAG) was used to pre-identify VCI -and COPD-related covariates. Variance Inflation Factor (VIF) eliminated highly collinear variables. Machine learning methods (LASSO, Random Forest, and XGBoost) screened variables. A weighted multivariate logistic regression model explored the VCI-COPD relationship. Restricted Cubic Spline (RCS) and threshold analysis examined non-linear relationships. Subgroup analysis and interaction tests ensured reliability. A nomogram showed the predictive factors’ importance for COPD. Model performance was reported using the Area Under the Receiver Operating Characteristic Curve (AUC).

**Results:**

In all models, we found that there was a negative correlation between VCI (≥50.1 mg/day) and the prevalence of COPD. The RCS and threshold analysis results show a negative correlation between COPD and VCI (≤135.6 mg/day). Subgroup analysis shows a negative association between VCI and the prevalence of COPD, specifically among females and individuals with dietary fiber intake in the second quartile (Q2). The AUC results show that our model has good diagnostic performance.

**Limitations:**

The cross-sectional design limits causal inference and lacks external validation.

**Conclusion:**

An elevated VCI within 50.1–135.6 is linked to a decreased risk for COPD.

## Highlights

- The relationship between vitamin C and COPD is not linear but exhibits a threshold effect. When vitamin C levels are 50.1–135.6 mg, it is negatively correlated with the risk of COPD.
- Vitamin C intake provides new guidance on preventing and treating COPD.
- LASSO + Random Forest + XGBoost shows excellent variable selection ability and avoids the overfitting problem in the model.
- Further clinical trials are needed to investigate the effectiveness and optimal range of vitamin C intake.

## 1 Introduction

Chronic Obstructive Pulmonary Disease (COPD) remains a leading cause of morbidity and mortality worldwide. According to the GOLD 2024 Report, COPD affects approximately 384 million people globally, with a prevalence of 10.3% among adults aged 40 years and older. It is the third leading cause of death worldwide, accounting for over 3.2 million deaths annually. The main features of COPD are persistent airway limitation and chronic inflammation in the airways and alveoli (1, 2). According to reports, from 2016 to 2020, the global number of COPD patients increased from 426 million to 467 million. It is projected that by 2025, the global number of COPD patients will reach around 530 million, with a Compound Annual Growth Rate (CAGR) of 2.7% for patient numbers from 2020 to 2025 (3, 4). In the United States, approximately 24 million people have airway limitations, among which about 16 million are diagnosed with COPD (5). The harms of COPD include respiratory failure (6), cardiovascular diseases (7, 8), psychological impacts (9, 10), sleep disorders (11), gastric ulcers (12, 13), spontaneous pneumothorax (14, 15), cor pulmonale (16, 17), severe impairment of lung function (18, 19), and so on, leading to a decline in the quality of life and a poor prognosis for patients. Thus, preventing COPD is crucial. Previous research has indicated that maintaining good lifestyle habits and a healthy diet can notably lower the occurrence rate of COPD (20). However, there is limited research available on the relationship between COPD and VCI per day.

VC helps with antioxidant effects, enhances the function of the immune system, has anti-inflammatory properties, promotes iron absorption, lowers blood hypertension, and improves vascular function and so on (21). For adults aged 19 years and older, the Recommended Dietary Allowance (RDA) for VC is 90 mg/day for men and 75 mg/day for women, according to the “Vitamin C—Health Professional Fact Sheet” of the National Institutes of Health (NIH) in the United States (22). Regrettably, in contemporary society, many people have difficulty getting enough Vitamin C Intake (VCI). This is because they often consume processed foods, have imbalanced diets, and may not prioritize fresh fruits and vegetables in their daily meals. Consequently, the prevalent practice of VC supplementation is gaining popularity. VC supplementation refers to increasing VCI by taking VC supplements or consuming vitamin C-rich foods to meet the body’s VC needs, especially for those who may not get enough VC from their daily diet. The relationship between COPD and VCI in the general population is still unclear. Figuring out this link can help us better understand how COPD and VCI interact. It may also have a positive impact on developing treatment strategies for COPD. This study aims to explore the possible connection between COPD and VCI among NHANES participants. The findings could offer better guidance for treating and preventing COPD.

## 2 Methods

### 2.1 Study population

**Figure 1:**
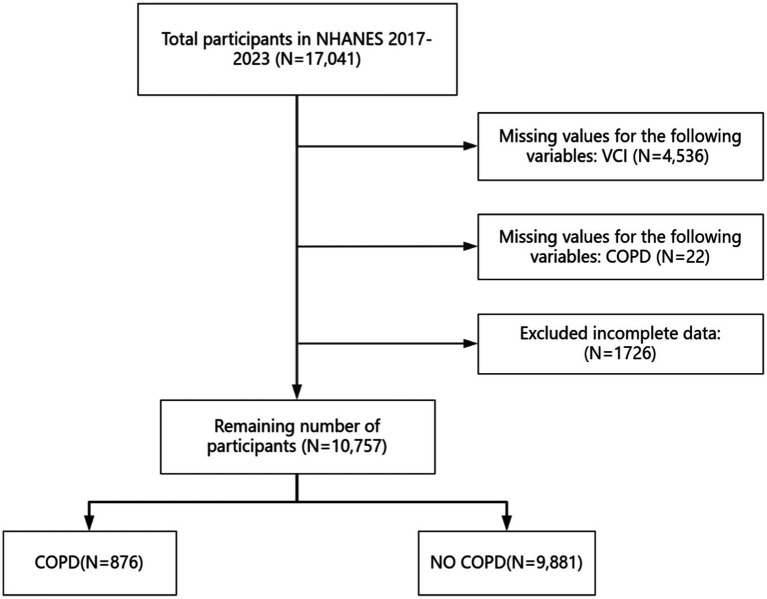
The flow chart of the included participants in this study.

The NHANES is a wide-ranging survey in the United States. It is overseen by the Centers for Disease Control and Prevention (CDC). The NHANES amasses cross-sectional data on the nutritional status and health of children and adults throughout the United States. It started in 1999 and conducts surveys every two years. The survey uses a sample that represents the entire nation. The objective of this initiative is to assess nutritional well-being and overall well-being among Americans aged 20 and above. The Institutional Review Board of the National Center for Health Statistics (NCHS) gave approval to this study. All participants provided written informed consent before taking part. We ensure that the entire research process adhered to relevant rules and guidelines. All research has adhered to the Declaration of Helsinki.^1^ This research incorporates information from two parts of the NHANES, spanning from 2017 to 2020 and from 2021 to 2023, involving a total of 17,041 participants. The criteria for participant exclusion in our study include: (1) lack of data on vitamin C intake; (2) absence of data regarding COPD; (3) surveys with missing information or incomplete data. The screening process is depicted in Figure 1.

### 2.2 The diagnosis of COPD

The diagnosis of COPD mainly depends on the existence of incompletely reversible airflow limitation. This is determined by a post-bronchodilator ratio of Forced Expiratory Volume in one second (FEV1) to Forced Vital Capacity (FVC) that is less than 0.7, measured using spirometry (23). FEV1 represents the volume of air an individual can exhale from their lungs within the first second following the deepest possible inhalation and is employed to gauge the airflow rate. Conversely, FVC refers to the maximum amount of air that can be exhaled following a full inhalation. It reflects the total ventilatory capacity of the lungs (24). A diminished FEV1/FVC ratio indicates the extent of airway obstruction in COPD patients. This ratio serves as one of the crucial markers for diagnosing COPD and is also highly significant for evaluating the severity of the disease and tracking its progression (25). In our article, we will solely select patients from the NHANES database diagnosed with COPD or those who deny having COPD for discussion.

### 2.3 Assessments of VCI

According to the serum test reports in the 2017–2023 NHANES surveys, data on VCI, defined as the total amount of VC that the human body consumes daily through diet, was acquired. The obtained VCI values ranged from 0–1977.4 mg (26). Subsequently, to better analyze this data, it was classified into four groups using quartile grouping: Q1 (0 ≤ VC < 19.3), Q2 (19.3 ≤ VC < 50.1), Q3 (50.1 ≤ VC < 110.6), Q4 (110.6 ≤ VC ≤ 1977.4). In this study, we analyzed data from participants’ serum test information and medical diagnosis documents, which have a certain degree of accuracy.

### 2.4 Covariates

We selected covariates based on existing literature and clinical experience, including gender (male or female); age (≥20 years); education (9–11th grade; college graduate or above; high school graduate; less than 9th grade; some college or AA degree); race (Mexican American; Non-Hispanic Asian; Non-Hispanic Black; Non-Hispanic White; Other Hispanic); smoke (yes or no); marital status (married/living with partner; never married; widowed/divorced/separated); drink (yes or no); BMI (14.6–92.3 kg/m^2^); hypertension (yes or no); diabetes (yes or no); dietary fiber (0–127.3 g); vitamin A (0–39,008 mcg); beta-carotene (0–71,772 mcg); vitamin K (0–2561.1 mcg); calcium (0–9,266 mg); potassium (0–14,358 mg); coronary heart disease (yes or no); malignancy (yes or no).

### 2.5 Statistical analyses

This research analyzed data with R statistical software (version 4.4.1) to study the link between VCI and COPD. Given the survey’s complexity, descriptive statistics were used on weighted data. The sample weights came from “WTMECPRP-Full sample 3 years MEC exam weight” (2017–2020) and “WTMEC2YR-Full sample MEC exam weight” (2021–2023). Continuous variables are presented as weighted means and standard deviations, and categorical variables are presented as weighted percentages. We compared categorical variables and continuous variables between different groups using the chi-square test and *t*-test, respectively. Using multiple imputations to handle missing covariate data. We used DAG to pre-identify covariates with relationships to VCI and COPD, excluding spurious associations. The VIF is used to eliminate variables with high collinearity. Machine learning (including LASSO, Random Forest, and XGBoost) was used to conduct variable screening, incorporate the selected data into multivariate logistic regression analysis, and explore the relationship between VCI and COPD after adjusting for confounding factors. Three models were established: Model 1 with no adjustments, Model 2 adjusting for selected covariates (gender, age, race, education, marital, drink, and BMI), and Model 3 adjusting for variables selected by machine learning (gender, age, education, marital, BMI, hypertension, diabetes, dietary fiber, vitamin A, vitamin K, calcium, potassium, coronary heart disease, malignancy, and smoke). Next, we further performed stratification and interaction analyses by all variables and plotted the associated forests. In addition, draw the RCS curve and threshold analysis to explore the nonlinear relationship between VCI and COPD. Then, a nomogram was constructed using the variables selected by machine learning to display the relative importance of these factors for predicting COPD. Finally, the diagnostic performance of the machine learning model and the full-variable model was evaluated using the AUC. All statistical tests were two-sided, and a *p*-value <0.05 was statistically significant.

## 3 Results

### 3.1 Description of participants’ basic information

This study comprised a sample of 10,757 individuals, reflecting approximately 0.003% of the American population. Table 1 presents the characteristics of participants. COPD is associated with various factors, including age, race, education, marital, drink, BMI, hypertension, diabetes, nutrient intake (dietary fiber, beta-carotene, vitamin C, vitamin K, calcium, and potassium), coronary heart disease, malignancies, and smoking habits. These factors can significantly influence the development of COPD.

**Table 1:** Characteristics of participants in the NHANES 2017–2023 cycles (*n* = 10,757).

Variable	Level	COPD		*p*-value
		No (*n* = 9,881)	Yes (*n* = 876)	
Gender	Female	5,229 (52.900)	474 (54.100)	0.522
	Male	4,652 (47.100)	402 (45.900)	
Age (years) mean (SD)		51.256 (17.295)	61.531 (14.145)	**<0.001**
Race	Mexican American	1,055 (10.700)	29 (3.300)	**<0.001**
	Non-Hispanic Asian	903 (9.100)	20 (2.300)	
	Non-Hispanic Black	2,187 (22.100)	185 (21.100)	
	Non-Hispanic White	4,686 (47.400)	574 (65.500)	
	Other Hispanic	1,050 (10.600)	68 (7.800)	
Education	9–11th grade	858 (8.700)	128 (14.600)	**<0.001**
	College graduate or above	3,184 (32.200)	111 (12.700)	
	High school graduate	2,189 (22.200)	268 (30.600)	
	Less than 9th grade	540 (5.500)	47 (5.400)	
	Some college or AA degree	3,110 (31.500)	322 (36.800)	
Marital	Married/Living with partner	5,729 (58.000)	419 (47.800)	**<0.001**
	Never married	1,966 (19.900)	131 (15.000)	
	Widowed/Divorced/Separated	2,186 (22.100)	326 (37.200)	
Drink	No	907 (9.200)	47 (5.400)	**<0.001**
	Yes	8,974 (90.800)	829 (94.600)	
BMI (kg/m^2^) mean (SD)		29.813 (7.169)	31.717 (9.125)	**<0.001**
Hypertension	No	6,334 (64.100)	355 (40.500)	**<0.001**
	Yes	3,547 (35.900)	521 (59.500)	
Diabetes	No	8,524 (86.300)	617 (70.400)	**<0.001**
	Yes	1,357 (13.700)	259 (29.600)	
Dietary fiber (g) mean (SD)		16.637 (10.601)	13.437 (8.794)	**<0.001**
Vitamin A (mcg) mean (SD)		593.571 (624.828)	611.650 (1405.880)	0.477
Beta-carotene (mcg) mean (SD)		2344.291 (4265.526)	2030.820 (4580.021)	**0.038**
VCI (mg)	Q1	2,414 (24.400)	286 (32.600)	**<0.001**
	Q2	2,446 (24.800)	241 (27.500)	
	Q3	2,490 (25.200)	194 (22.100)	
	Q4	2,531 (25.600)	155 (17.700)	
Vitamin K (mcg) mean (SD)		124.583 (176.176)	103.787 (134.309)	**0.001**
Calcium (mg) mean (SD)		898.833 (563.670)	849.705 (583.464)	**0.014**
Potassium (mg) mean (SD)		2522.758 (1267.217)	2356.852 (1295.346)	**<0.001**
Coronary heart disease	No	9,504 (96.200)	735 (83.900)	**<0.001**
	Yes	377 (3.800)	141 (16.100)	
Malignancy	No	8,720 (88.300)	689 (78.700)	**<0.001**
	Yes	1,161 (11.700)	187 (21.300)	
Smoke	No	6,116 (61.900)	227 (25.900)	**<0.001**
	Yes	3,765 (38.100)	649 (74.100)	

VCI, vitamin C intake: Q1 (0 ≤ VCI < 19.3), Q2 (19.3 ≤ VCI < 50.1), Q3 (50.1 ≤ VCI < 110.6), Q4 (110.6 ≤ VCI ≤ 1977.4). The bold values are highlight statistically significant indicators that were clearly pointed out during revision in response to reviewers’ requests (e.g., *p* < 0.05) and represent key findings in our analysis.

### 3.2 Multiple imputation

**Figure 2:**
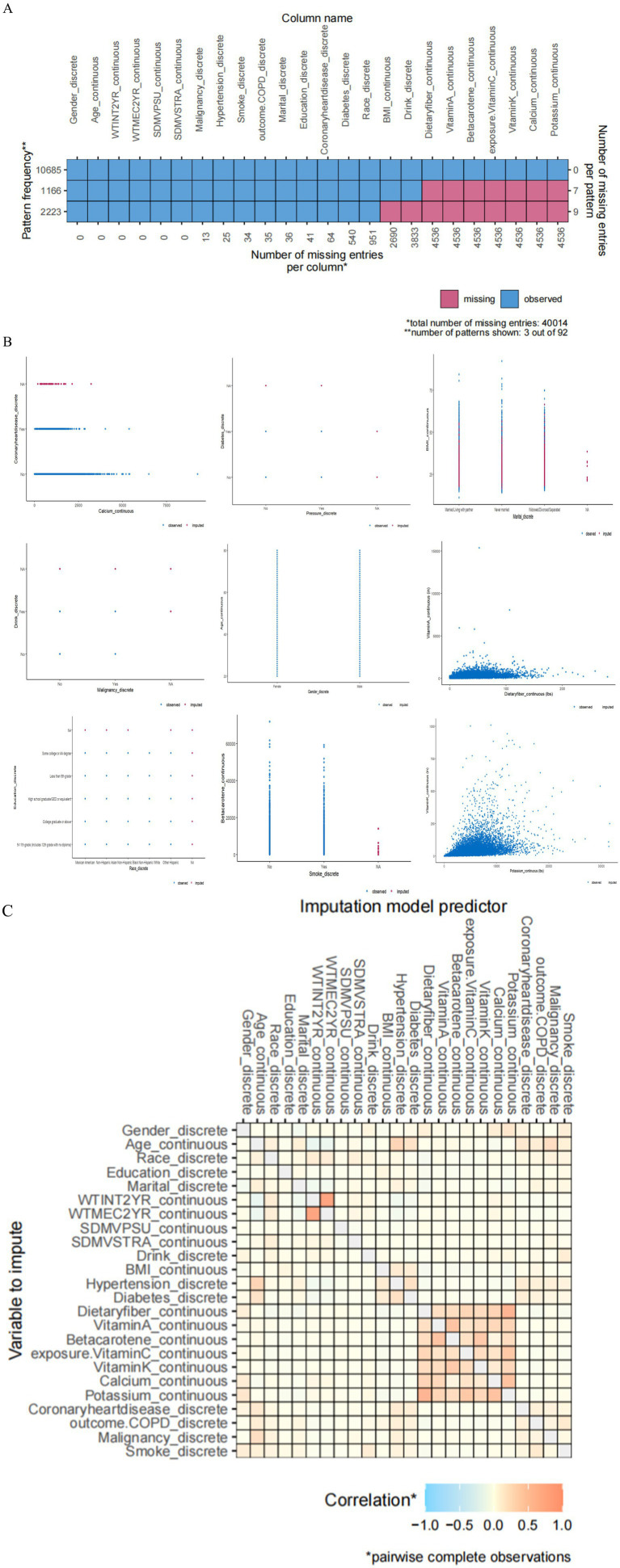
**(A)** Missing data pattern plot. **(B)** In the plot of the imputed data, blue denotes observed data, and red signifies imputed data. **(C)** A heatmap of the correlations among covariates.

We performed imputation using the mice package and utilized the ggmice and ggplot2 packages for plotting. After filtering the exposure and outcome variables, we set the seed to 111. Regarding the missing data in the original dataset, for BMI, there are 2,690 missing values, accounting for 25.04%; for drink, 3,833 missing values make up 18.94%. Dietary fiber has 4,536 missing values with a proportion of 16.81%, while vitamin A, beta-carotene, vitamin C, vitamin K, calcium, and potassium all have 4536 missing values, each at a proportion of 26.62%. From Figure 2A, it’s clear that variables such as “dietary fiber,” “vitamin A,” “beta-carotene,” “vitamin C,” “vitamin K,” and “calcium” have a significant number of missing values, as indicated by the prominent red portions of the bars in the chart. Figure 2B is a visualization of the missing data from various variables, categorized into continuous and discrete data. In the heatmap from Figure 2C, we can observe the correlation of missing data patterns between different variables. The legend shows the correspondence between the color and the correlation coefficient, where blue represents a negative correlation, orange indicates a positive correlation, and the deeper the color, the stronger the correlation. It can be seen that there is a strong correlation among dietary indicators, and a significant correlation is also observed between calcium and potassium. We have included the following in the supplementary file for your reference: ① The sample size and missing values at each step of the data processing in this article. ② The relevant figures and tables of the covariates (including vitamin D and magnesium); ③ A summary table of all the abbreviations (Supplementary material).

### 3.3 Covariate selection by machine learning

**Figure 3:**
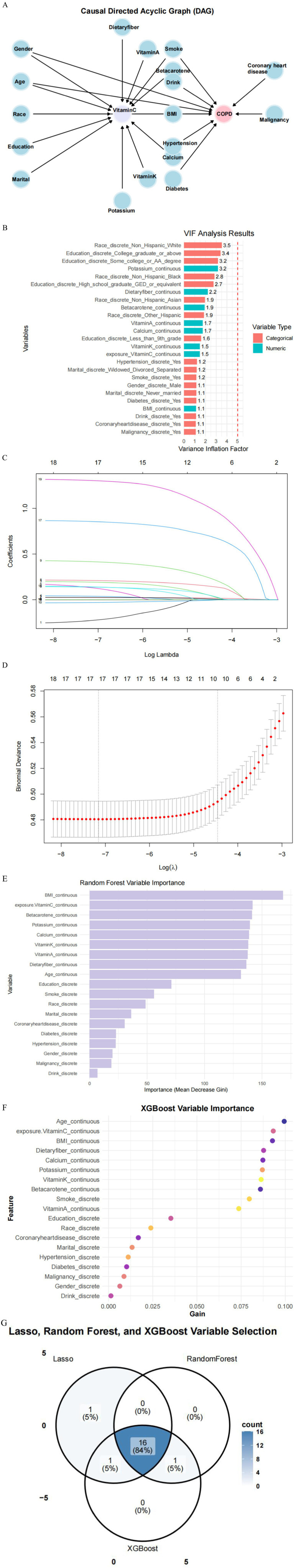
**(A)** Directed Acyclic Graph (DAG) for pre-identifying covariates. **(B)** Conduct Variance Inflation Factor (VIF) detection for all variables. **(C)** LASSO regression path plot. **(D)** LASSO 10-fold cross-validation plot. **(E)** Random Forest variable importance plot. **(F)** Variable importance plot for an XGBoost model. **(G)** Three algorithmic Venn diagram screening variables.

Before establishing a machine learning model, to ensure the accuracy and reliability of the model, first, we used DAG to pre-identify covariates with plausible causal relationships to VCI and COPD, excluding spurious associations (Figure 3A). Then, we use the VIF to evaluate the degree of collinearity between variables. By identifying and excluding variables with a VIF >5 value, we can simplify the model structure, reduce unnecessary variables. As shown in Figure 3B, there is no multicollinearity among our variables.

LASSO regression is particularly effective in handling multicollinearity by shrinking the coefficients of correlated predictors, thereby reducing overfitting and improving model interpretability. This is a significant advantage over traditional linear regression, which can produce unstable estimates in the presence of multicollinearity. We obtained the model coefficients at the best lambda value using coef(cv_lasso, s = “lambda.min”). The output shows a sparse matrix with some coefficients being zero, indicating that these variables are excluded in the optimal model. Through computation, we obtained the variables selected by Lasso, as follows: gender, age, race, education, marital, drink, BMI, hypertension, diabetes, dietary fiber, vitamin A, vitamin C, vitamin K, calcium, potassium, coronary heart disease, malignancy, and smoke (Figures 3C,D).

Random Forest is a classification algorithm composed of multiple decision trees. It constructs machine-learning models by randomly sampling training data and determining the optimal splitting approach. In Random Forest, each decision tree uses feature metrics that match the dataset’s characteristics to assess the significance of each feature. The Random Forest algorithm generated 500 trees, and each split of the decision tree randomly selected 18 predictive variables (BMI, vitamin C, beta-carotene, potassium, calcium, vitamin K, vitamin A, dietary fiber, age, education, smoke, race, marital, coronary heart disease, diabetes, hypertension, gender, and malignancy) (Figure 3E).

XGBoost is an advanced ensemble learning method that builds classification trees through iterative boosting. We implemented XGBoost with the following specifications: 100 training rounds (*n*rounds), binary logistic objective function, default learning rate (*η* = 0.3), maximum tree depth of 6. The algorithm combines multiple weak classifiers into a strong predictive model through additive training, creating an interconnected decision tree structure ideal for classification tasks. Our implementation demonstrated excellent generalization capability and scalability (27), ultimately identifying 18 predictor variables (age, vitamin C, BMI, dietary fiber, calcium, potassium, vitamin K, beta-carotene, smoke, vitamin A, education, race, coronary heart disease, marital, hypertension, diabetes, malignancy, and gender). Figure 3F shows the importance of these variables.

Finally, we used a Venn diagram (Figure 3G) to show that 16 variables (gender, age, education, marital, BMI, hypertension, diabetes, dietary fiber, vitamin A, vitamin C, vitamin K, calcium, potassium, coronary heart disease, malignancy, and smoke) that overlapped each other were found through the intersection of the three machine learning algorithms described above.

### 3.4 The models of VCI and COPD

After performing a weighted multivariate logistic regression analysis (Table 2), our results indicate that a higher VCI is associated with an decreased risk of developing COPD. In the unadjusted model, groups with higher vitamin C intake (Q3, Q4) all showed a trend of reduced COPD risk. After adjusting for more confounding factors, the associations for Q3 and Q4 remained significant, indicating a stable relationship between higher VCI (≥50.1 mg) and reduced COPD risk. Compared with the lowest quartile, the risk of developing COPD in the highest quartile decreased by 51% in the Model 1 (OR = 0.490; 95% CI = 0.357–0.672, *p* = 0.000), 43% in the Model 2 (OR = 0.566; 95% CI = 0.413–0.776, *p* = 0.002) and 44% in the Model 3 (OR = 0.562; 95% CI = 0.380–0.832, *p* = 0.010).

**Table 2:** Multivariate logistic regression analysis of the linkage between VCI and COPD.

Total VCI quantile	Model 1		Model 2		Model 3	
	OR (95% CI)	*p*-value	OR (95% CI)	*p*-value	OR (95% CI)	*p*-value
Q1	ref		ref		ref	
Q2	0.758 (0.567–1.011)	0.068	0.782 (0.583–1.049)	0.114	0.830 (0.609–1.130)	0.253
Q3	0.635 (0.497–0.812)	**0.001**	0.705 (0.544–0.914)	**0.015**	0.706 (0.526–0.949)	**0.033**
Q4	0.490 (0.357–0.672)	**0.000**	0.566 (0.413–0.776)	**0.002**	0.562 (0.380–0.832)	**0.010**

CI, confidence interval; OR, odds ratio. Model 1: Unadjusted. Model 2: Adjusted for gender, age, race, education, marital, drink, and BMI. Model 3: Adjusted for gender, age, education, marital, BMI, hypertension, diabetes, dietary fiber, vitamin A, vitamin K, calcium, potassium, coronary heart disease, malignancy, and smoke. (Variables screened by three machine learning methods). The bold values are highlight statistically significant indicators that were clearly pointed out during revision in response to reviewers’ requests (e.g., *p* < 0.05) and represent key findings in our analysis.

### 3.5 Subgroup, interaction analyses and forest plot

**Figure 4:**
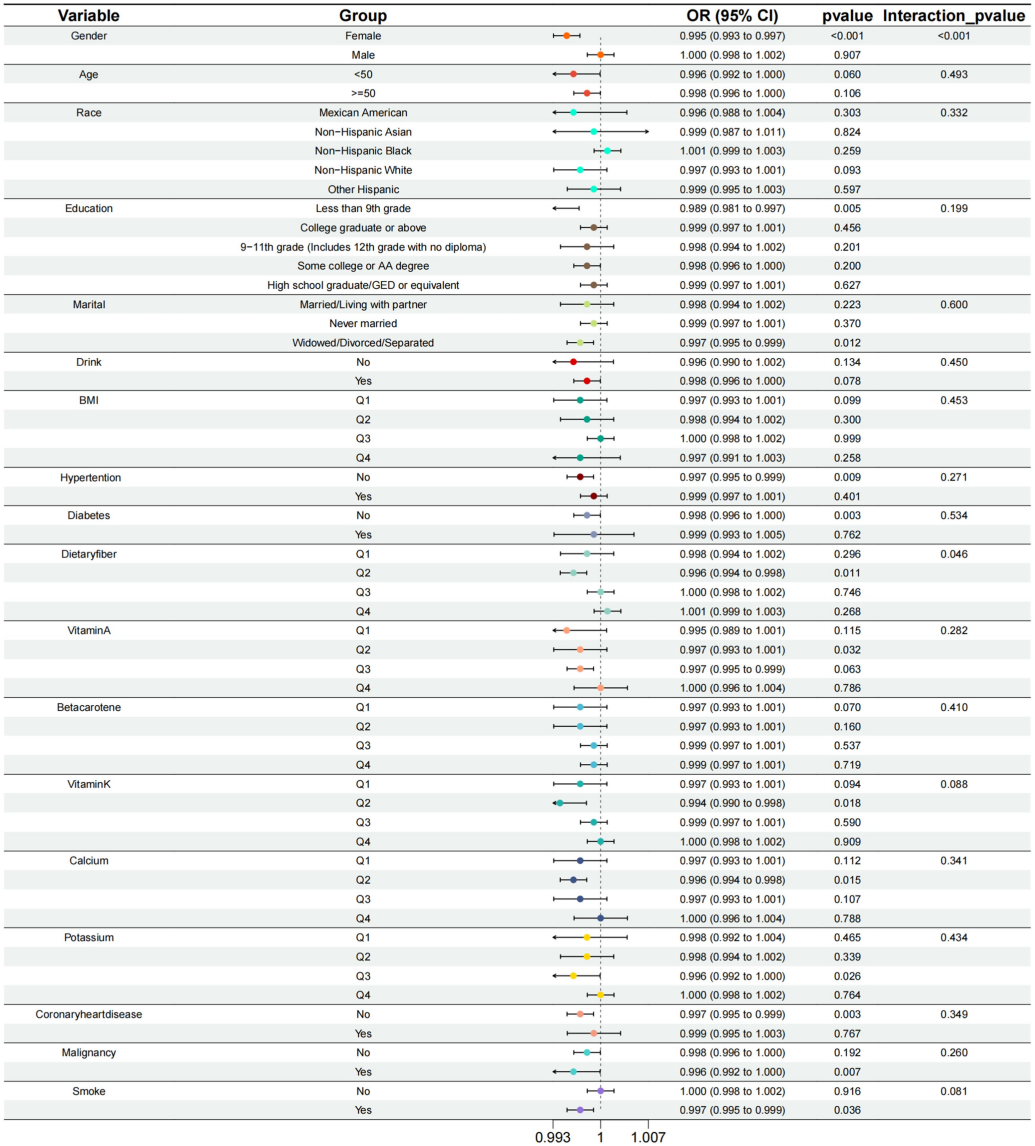
Subgroup analysis of the relationship between calcium intake and COPD risk at different levels.

To avoid missing interaction terms, we conducted subgroup analyses for all variables. The subgroups were based on four different quartiles of VCI. By the results of the subgroup analysis, we find that the effect of VCI on reducing the risk of COPD is more pronounced in specific groups of people. These groups include females and those with second-quartile (Q2) dietary fiber intake (Table 3 and Figure 4).

**Table 3:** Hierarchical analysis and interaction effects.

Variable	Group	Estimate	Std. error	*t* value	*p*-value	*p* for interaction
Gender	Female	−0.005	0.001	−4.430	**0.000**	**0.000**
	Male	0.000	0.001	−0.117	0.907	
Age	<50	−0.004	0.002	−1.941	0.060	0.493
	≥50	−0.002	0.001	−1.657	0.106	
Race	Mexican American	−0.004	0.004	−1.051	0.303	0.332
	Non-Hispanic Asian	−0.001	0.006	−0.224	0.824	
	Non-Hispanic Black	0.001	0.001	1.145	0.259	
	Non-Hispanic White	−0.003	0.002	−1.721	0.093	
	Other Hispanic	−0.001	0.002	−0.533	0.597	
Education	Less than 9th grade	−0.011	0.004	−2.971	0.005	0.199
	College graduate or above	−0.001	0.001	−0.753	0.456	
	9–11th grade (includes 12th grade with no diploma)	−0.002	0.002	−1.299	0.201	
	Some college or AA degree	−0.002	0.001	−1.304	0.200	
	High school graduate/GED or equivalent	−0.001	0.001	−0.490	0.627	
Marital	Married/Living with partner	−0.002	0.002	−1.237	0.223	0.600
	Never married	−0.001	0.001	−0.906	0.370	
	Widowed/Divorced/Separated	−0.003	0.001	−2.634	0.012	
Drink	No	−0.004	0.003	−1.532	0.134	0.450
	Yes	−0.002	0.001	−1.810	0.078	
BMI	Q1 (14.6–24.8)	−0.003	0.002	−1.690	0.099	0.453
	Q2 (24.8–28.7)	−0.002	0.002	−1.050	0.300	
	Q3 (28.7–33.7)	0.000	0.001	−0.002	0.999	
	Q4 (33.7–92.3)	−0.003	0.003	−1.147	0.258	
Hypertension	No	−0.003	0.001	−2.760	0.009	0.271
	Yes	−0.001	0.001	−0.850	0.401	
Diabetes	No	−0.002	0.001	−3.110	0.003	0.534
	Yes	−0.001	0.003	−0.305	0.762	
Dietary fiber	Q1 (0.0–9.2)	−0.002	0.002	−1.059	0.296	**0.046**
	Q2 (9.2–14.3)	−0.004	0.001	−2.658	**0.011**	
	Q3 (14.3–21.1)	0.000	0.001	0.327	0.746	
	Q4 (21.1–127.3)	0.001	0.001	1.124	0.268	
Calcium	Q1 (0–510)	−0.003	0.002	−1.625	0.112	0.341
	Q2 (510–782)	−0.004	0.001	−2.551	0.015	
	Q3 (782–1,140)	−0.003	0.002	−1.652	0.107	
	Q4 (1,140–9,266)	0.000	0.002	−0.271	0.788	
Vitamin A	Q1 (0–250)	−0.005	0.003	−1.611	0.115	0.282
	Q2 (250–456)	−0.003	0.002	−2.222	0.032	
	Q3 (456–752)	−0.003	0.001	−1.913	0.063	
	Q4 (752–39,008)	0.000	0.002	−0.274	0.786	
Vitamin K	Q1 (0.0–40.8)	−0.003	0.002	−1.717	0.094	0.088
	Q2 (40.8–74.9)	−0.006	0.002	−2.461	0.018	
	Q3 (74.9–137.7)	−0.001	0.001	−0.543	0.590	
	Q4 (137.7–2561.1)	0.000	0.001	−0.115	0.909	
Potassium	Q1 (0–1,645)	−0.002	0.003	−0.739	0.465	0.434
	Q2 (1,645–2,313)	−0.002	0.002	−0.967	0.339	
	Q3 (2,313–3,129)	−0.004	0.002	−2.319	0.026	
	Q4 (3,129–14,358)	0.000	0.001	−0.302	0.764	
Coronary heart disease	No	−0.003	0.001	−3.119	0.003	0.349
	Yes	−0.001	0.002	−0.298	0.767	
Malignancy	No	−0.002	0.001	−1.326	0.192	0.260
	Yes	−0.004	0.002	−2.825	0.007	
Smoke	No	0.000	0.001	0.106	0.916	0.081
	Yes	−0.003	0.001	−2.166	0.036	
Beta-carotene	Q1 (0–272)	−0.003	0.002	−1.860	0.070	0.410
	Q2 (272–814)	−0.003	0.002	−1.433	0.160	
	Q3 (814–2,529)	−0.001	0.001	−0.624	0.537	
	Q4 (2,529–71,772)	−0.001	0.001	−0.363	0.719	

Each variable was adjusted according to every element and then analyzed. Divide all numeric variables into quartiles while keeping categorical variables unchanged. The bold values are highlight statistically significant indicators that were clearly pointed out during revision in response to reviewers’ requests (e.g., *p* < 0.05) and represent key findings in our analysis.

### 3.6 Non-linear relationship and threshold analysis results

**Figure 5:**
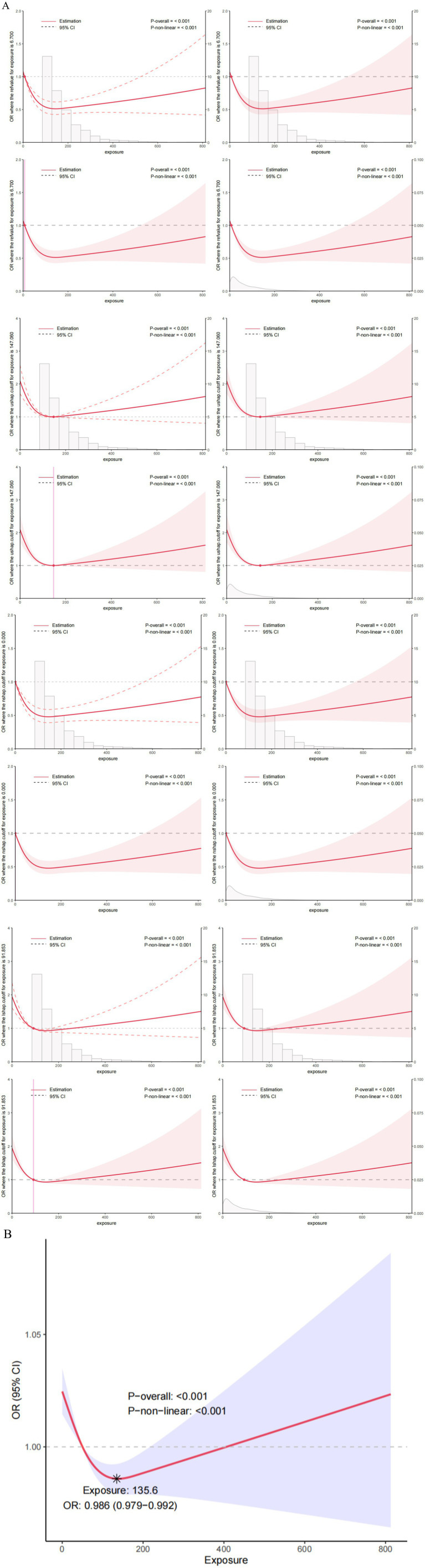
**(A)** The Restricted Cubic Splines (RCS) curve shows the association between VCI and COPD in all study participants. We did not conduct variable adjustments. **(B)** Nonlinear relationship between VCI and COPD: restricted cubic spline analysis, with node at 135.6 mg. In the RCS regression, adjustments were made for gender, age, education, marital, BMI, hypertension, diabetes, dietary fiber, vitamin A, vitamin K, calcium, potassium, coronary heart disease, malignancy, and smoke.

First, we constructed the RCS curve without covariates. *p*-overall value of <0.001 shows the overall model is statistically significant. This means there’s a significant link between VCI and the risk of COPD. *p*-non-linear: <0.001 indicates that this association has a significant non-linear characteristic, i.e., the relationship is not a simple straight-line association (Figure 5A). Then, we constructed the RCS using the comprehensive variables screened by machine learning, as shown in Figure 5B. There is a threshold effect in the association between the continuous variable of VCI and the discrete variable of COPD (*p* for likelihood test <0.001). No association was found between VCI and COPD when the number of VCI was exceed 135.605. However, when the number of VCI ≤135.605, a negative association was observed between VCI and COPD (OR = 0.996; 95% CI = 0.993–0.998, *p* = 0.002) (Table 4).

**Table 4:** Threshold analysis result.

Outcome	Effect	*p*-value
Model 1 Fitting model by standard linear regression	0.999 (0.998–1.000)	0.182
Model 2 Fitting model by two-piecewise linear regression		
Inflection point	135.601	
<135.601	0.996 (0.993–0.998)	**0.002**
≥135.601	1.001 (0.999–1.002)	0.243
*p* for likelihood test		**<0.001**

Adjusted for gender, age, education, marital, BMI, hypertension, diabetes, dietary fiber, vitamin A, vitamin K, calcium, potassium, coronary heart disease, malignancy, and smoke. The bold values are highlight statistically significant indicators that were clearly pointed out during revision in response to reviewers’ requests (e.g., *p* < 0.05) and represent key findings in our analysis.

### 3.7 Evaluation of the nomogram model

**Figure 6:**
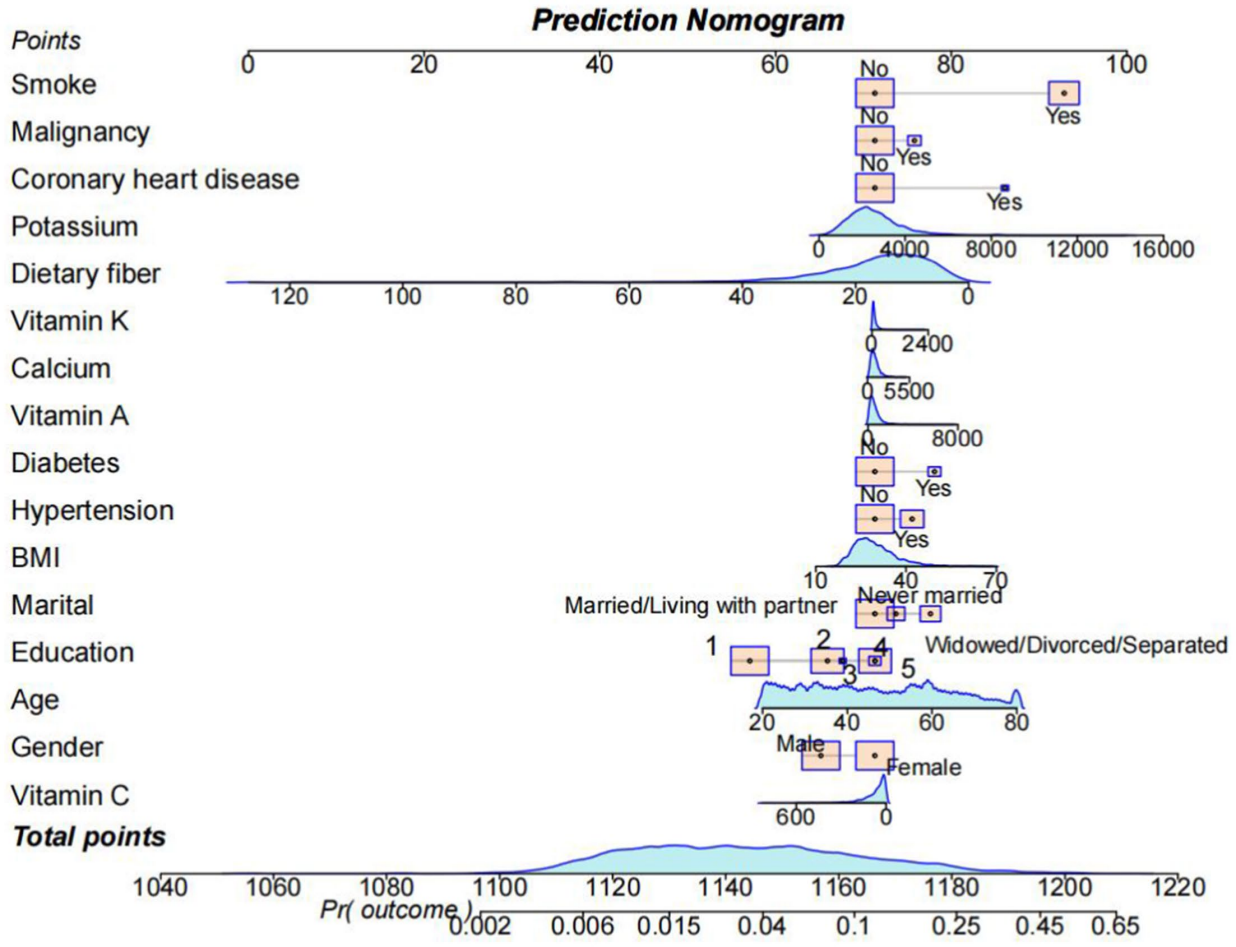
A nomogram was used to estimate the risk of COPD related to VCI. First, sum up the points of each feature to get the total points. Then, draw a vertical line at the total points to determine the corresponding “risk of COPD.” (Education) College graduate or above: 1; Some college or AA degree: 2; High school graduate/GED or equivalent: 3; Less than 9th grade: 4; 9-11th grade (Includes 12th grade with no diploma): 5.

Based on the above three machine learning methods, we screened out 16 variables and constructed a nomogram to predict the diverse trajectories associated with the risk of developing COPD, as shown in Figure 6. Each factor was assigned a score on the point scale axis. By adding up these individual scores, we could calculate a total score. Then, by mapping this total score onto the bottom risk scale axis, we could estimate the probability of different trajectories in the development of COPD.

#### 3.8 Machine learning and all-variables model predictions

**Figure 7:**
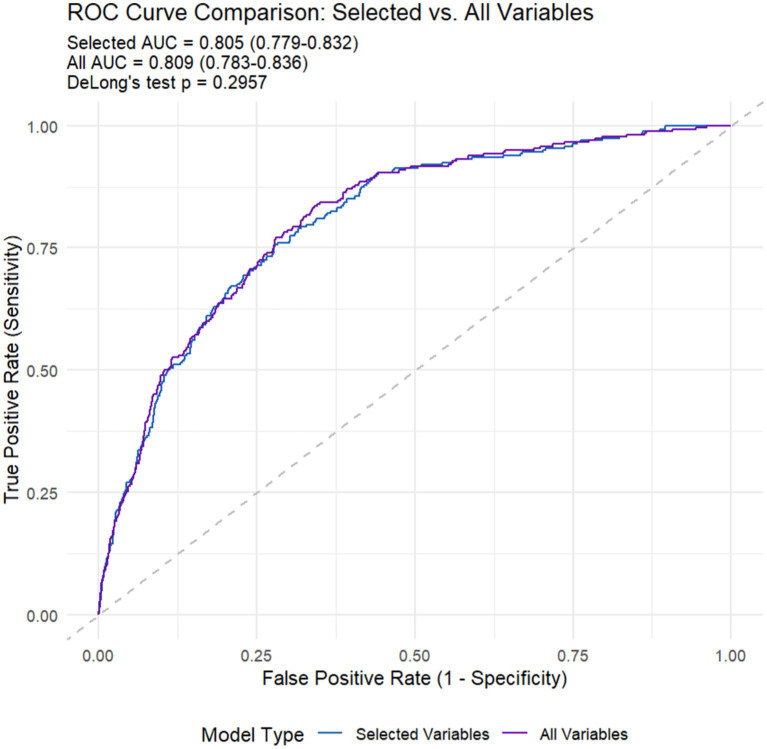
The Receiver Operating Characteristic (ROC) curves of machine learning (selected variables) and all variables models.

As presented in Table 5 and Figure 7, the all-variables model had an AUC of 0.809, along with a sensitivity of 73.3% and a specificity of 76.5%. The machine learning model showed an AUC of 0.805, with a sensitivity of 72.9% and a specificity of 76.0%. All models demonstrated good predictive value. Since DeLong’s *p*-value is 0.2957 > 0.05, we can conclude that there is no difference in the diagnostic capabilities between the two models. Therefore, machine learning has helped us screen out the fewest and optimal diagnostic variables.

**Table 5:** Sensitivity analysis.

Model performance metrics					
Model	AUC	AUC (95% CI)	Sensitivity	Specificity	DeLong’s *p*
Selected variables	**0.805**	**0.805 (0.779–0.832)**	72.9%	76.0%	**0.296**
All variables	**0.809**	**0.809 (0.783–0.836)**	73.3%	76.5%	

Comparison of the sensitivity and specificity between the machine learning model and the all-variables model. The bold values are highlight statistically significant indicators that were clearly pointed out during revision in response to reviewers’ requests (e.g., *p* < 0.05) and represent key findings in our analysis.

## 4 Discussion

Before researching the risk of COPD, understanding the importance of the relationships between dietary nutrients and chronic diseases is essential. In this study, we investigated the link between the VCI and COPD prevalence, utilizing data sourced from the NHANES database. The findings indicated a significant negative link between VCI and a greater risk of COPD. Firstly, in the weighted logistic regression analysis, regardless of whether covariates were unadjusted, adjusted for demographic variables, or fully adjusted, we consistently found that there was a significant negative association between VCI (≥50.1 mg/day) and the prevalence of COPD. Additionally, in the RCS curve and the corresponding threshold effect analysis, we observed a nonlinear relationship between the VCI and COPD, with an inflection point at 135.6 mg. When the VCI is under 135.6 mg, with the increase in the intake of VCI, the risk of COPD may decrease. This is consistent with the results demonstrated by the weighted logistic regression, further validating the reliability of our conclusion. It indicates that when VC levels are 50.1–135.6 mg, an increase in vitamin C intake is significantly associated with a reduction in the risk of COPD. To derive more accurate insights, after conducting subgroup analysis, we observed a negative association between VCI and the prevalence of COPD, specifically among females and individuals with dietary fiber intake in the second quartile (Q2). This indicates that keeping VCI at an optimal level is crucial for lung health. It may also be associated with the prevention of COPD.

Before screening variables, we determined the correlations of covariates using DAG diagrams and detected multicollinearity by employing the VIF. Then, we employed LASSO, Random Forest, and XGBoost to screen for important variables, reduce the risk of overfitting, and simplify the model. The advantage of this combination lies in integrating LASSO’s feature selection capacity, Random Forest’s proficiency in handling nonlinear relationships, and XGBoost’s robust prediction capabilities. In previous studies, machine learning has been used extensively in the clinical prediction of sepsis in ICU patients (27), acute kidney injury (28), and so on. Other machine learning also uses blood heavy metal data to predict COPD (29, 30). However, similar studies have rarely focused on diet and have only considered the effect of a single aspect on COPD.

To our knowledge, this is the first study based on NHANES data from 2017 to 2023 that assesses the association between VCI and COPD risk. Compared to previous studies that primarily used older NHANES datasets, these studies mainly investigated the relationship between common health indicators or specific nutrients and diseases. For instance, research has indicated that an excessive intake of vitamin C supplements may cause acute renal failure. Consequently, individuals should exercise caution regarding their vitamin C intake (31). VC, also called ascorbate or L-ascorbic acid, acts as an antioxidant. It’s important for the immune system, involved in allergic reactions, keeping connective tissue healthy, and even suppressing tumors (32–34). Low VC levels are linked to more wheezing, shortness of breath, and worse COPD symptoms (35–37). Eating foods rich in VC can reduce oxidative stress, boost collagen production, and bring back normal levels of vascular endothelial growth factor and the growth of lung alveolar cells (38). Many studies (39–40) have found that getting enough VCI can help prevent COPD. Our results support these findings. The results of this study are extremely important as it represents a vast majority of the U.S. population. Previously, Park et al. (41) revealed the impact of dietary antioxidants on COPD in South Korea. Our study, with a large amount of data, strongly supports this previous research. Many researchers have attempted to verify using NHANES data. However, numerous such studies overlooked the complex survey design. This oversight can lead to biased results and overstate the significance level. In contrast, we adhered to the guidelines recommended by the institutions conducting this complex survey. As a result, the finding regarding the positive effect of VCI on COPD is considered reliable.

The interplay between VCI and COPD is governed by intricate factors. VC is a potent antioxidant that neutralizes Reactive Oxygen Species (ROS) generated by cigarette smoke and environmental pollutants. COPD is characterized by chronic oxidative stress, which damages lung tissue and perpetuates inflammation (42). VC modulates immune responses by inhibiting pro-inflammatory cytokines (e.g., IL-6, TNF-α) and enhancing neutrophil apoptosis, which is dysregulated in COPD (43). This association requires further verification through more in-depth longitudinal studies.

However, our research has some significant limitations. First, we used a cross-sectional research method. So, we cannot accurately figure out if there’s a cause- and effect link between VCI and the prevalence of COPD. Second, although we considered many potential confounding factors, there could still be unknown factors influencing the results. Third, in this study, we only divided the participants into the COPD group and the non-COPD group. We did not do more detailed analyses of pulmonary function, disease grading, and occupational exposure.

Our study used secondary data from NHANES. These data are useful, but they have problems. Some important variables might be missing or not defined clearly because the data were not collected specifically for our study. We could not control how the data were collected, which might lead to biases or measurement mistakes. Also, the data’s timeliness might limit how well our findings can be applied more widely. Future research could collect primary data or use data from different sources to confirm our results.

## 5 Conclusion

This large-scale national study demonstrates that dietary VCI is protective against COPD independent of smoking history in the American general population. Maintaining a reasonable daily intake of VC may serve as a practical preventive strategy for COPD. However, VC supplementation should not replace smoking cessation or other evidence-based therapies, but rather complement existing preventive and therapeutic approaches for COPD.

## Data Availability

The original contributions presented in the study are included in the article/Supplementary material, further inquiries can be directed to the corresponding author.

## Footnotes

^1^ https://www.cdc.gov/nchs/nhanes/index.htm
