# Supplementary material for: Relationships between vitamin C intake and COPD assessed by machine learning approaches from the NHANES (2017–2023)
Source: Front Nutr. 2025 May 15;12:1563692. doi: 10.3389/fnut.2025.1563692 (PMC12119308; doi:10.3389/fnut.2025.1563692)
Supplement: Supplementary file 1 [file Table_1.docx]

The Supplementary Document contains items ①, ② and ③

1. The following is from the original manuscript (excluding the supplements of magnesium and vitamin D)

Supplementary Table 1

| Term | Number | Missing data step by step |
| --- | --- | --- |
| All | 17041 |  |
| exposure.VitaminC | 12505 | -4536 |
| outcome.COPD | 12483 | -22 |
| Gender | 12483 | -0 |
| Age | 12483 | -0 |
| Race | 11819 | -664 |
| Education | 11811 | -8 |
| Marital | 11806 | -5 |
| WTINT2YR | 11806 | -0 |
| WTMEC2YR | 11806 | -0 |
| SDMVPSU | 11806 | -0 |
| SDMVSTRA | 11806 | -0 |
| Drink | 11197 | -609 |
| BMI | 11197 | -0 |
| Pressure | 11187 | -10 |
| Diabetes | 10834 | -353 |
| Dietaryfiber | 10834 | -0 |
| VitaminA | 10829 | -5 |
| Betacarotene | 10824 | -5 |
| VitaminK | 10824 | -0 |
| Calcium | 10812 | -12 |
| Potassium | 10806 | -6 |
| Coronaryheartdisease | 10772 | -34 |
| Malignancy | 10767 | -5 |
| Smoke | 10757 | -10 |

Notes: This is the sample quantity and the situation of missing values at each step during the data processing after filtering the exposure variables and outcome variables.

② The following is an analysis of the key parts that include magnesium and vitamin D.

Figure S1

**
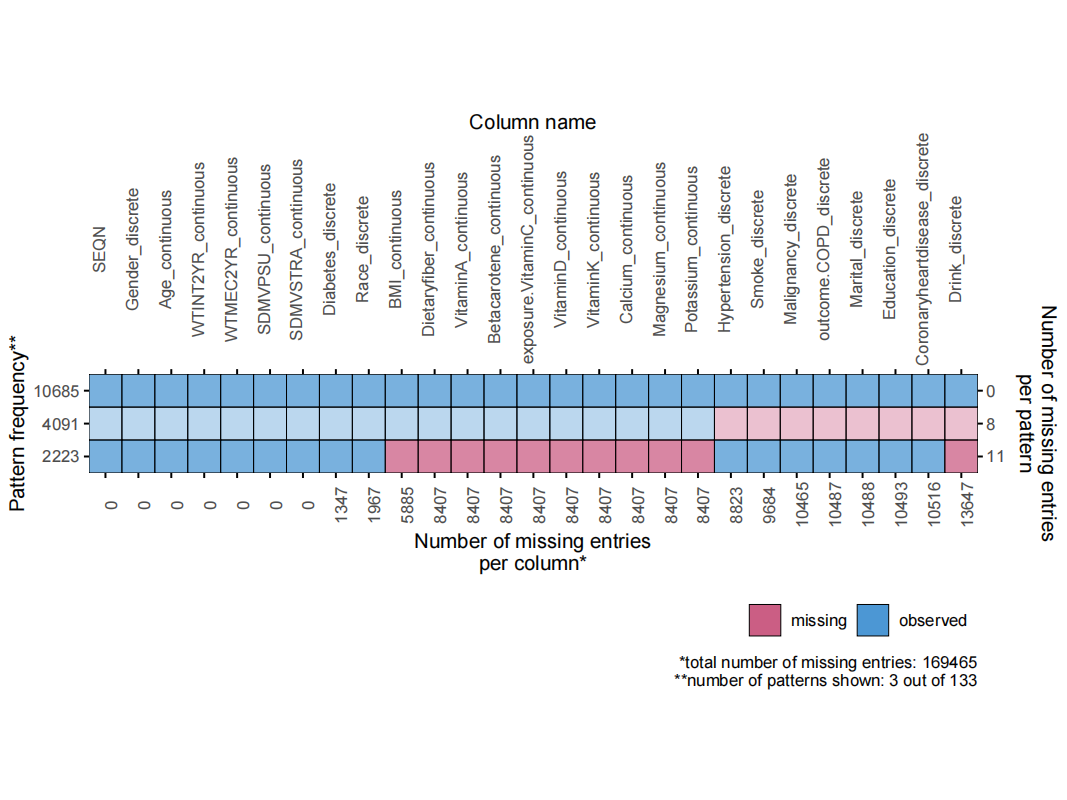
**

Missing data pattern plot (with added VitaminD and Magnesium).

Supplementary Table 2

| Term | Number |
| --- | --- |
| All | 27493 |
| exposure.VitaminC | 19086 |
| outcome.COPD | 12483 |
| Gender | 12483 |
| Age | 12483 |
| Race | 11819 |
| Education | 11811 |
| Marital | 11806 |
| WTINT2YR | 11806 |
| WTMEC2YR | 11806 |
| SDMVPSU | 11806 |
| SDMVSTRA | 11806 |
| Drink | 11197 |
| BMI | 11197 |
| Hypertension | 11187 |
| Diabetes | 10834 |
| Dietaryfiber | 10834 |
| VitaminA | 10829 |
| Betacarotene | 10824 |
| VitaminD | 10824 |
| VitaminK | 10824 |
| Calcium | 10812 |
| Magnesium | 10811 |
| Potassium | 10805 |
| Coronaryheartdisease | 10771 |
| Malignancy | 10766 |
| Smoke | 10756 |

Notes: This is the sample quantity and the situation of missing values at each step during the data processing after filtering the exposure variables and outcome variables (with added VitaminD and Magnesium).

Supplementary Table 3

Characteristics of participants in the NHANES 2017 - 2023 cycles (n = 10757).

| Variable | level | COPD | | P-value |
| --- | --- | --- | --- | --- |
|  |  | No (n=9880) | Yes (n=876) |  |
| Gender.... | Female | 5229 (52.9) | 474 (54.1) | 0.522 |
|  | Male | 4651 (47.1) | 402 (45.9) |  |
| Age(years)..mean..SD.., |  | 51.256 (17.295) | 61.531 (14.145) | **<0.001** |
| Race.... | Mexican American | 1055 (10.700) | 29 (3.300) | **<0.001** |
|  | Non-Hispanic Asian | 903 (9.100) | 20 (2.300) |  |
|  | Non-Hispanic Black | 2187 (22.100) | 185 (21.100) |  |
|  | Non-Hispanic White | 4686 (47.400) | 574 (65.500) |  |
|  | Other Hispanic | 1050 (10.600) | 68 ( 7.800) |  |
| Education.... | 9-11th grade | 858 ( 8.700) | 128 (14.600) | **<0.001** |
|  | College graduate or above | 3184 (32.200) | 111 (12.700) |  |
|  | High school graduate | 2189 (22.200) | 268 (30.600) |  |
|  | Less than 9th grade | 540 ( 5.500) | 47 ( 5.400) |  |
|  | Some college or AA degree | 3110 (31.500) | 322 (36.800) |  |
| Marital.... | Married/Living with partner | 5728 (58.000) | 419 (47.800) | **<0.001** |
|  | Never married | 1966 (19.900) | 131 (15.000) |  |
|  | Widowed/Divorced/Separated | 2186 (22.100) | 326 (37.200) |  |
| Drink.... | No | 907 ( 9.200) | 47 ( 5.400) | **<0.001** |
|  | Yes | 8974 (90.800) | 829 (94.600) |  |
| BMI(kg/m2)..  mean..SD.. |  | 29.813 (7.169) | 31.717 (9.125) | **<0.001** |
| Hypertension.... | No | 6334 (64.100) | 355 (40.500) | **<0.001** |
|  | Yes | 3547 (35.900) | 521 (59.500) |  |
| Diabetes.... | No | 8524 (86.300) | 617 (70.400) | **<0.001** |
|  | Yes | 1357 (13.700) | 259 (29.600) |  |
| Dietary fiber (g)..mean..SD.. |  | 16.637 (10.601) | 13.437 (8.794) | **<0.001** |
| Vitamin A (mcg)..mean..SD.. |  | 593.571 (624.828) | 611.650 (1405.880) | 0.477 |
| Beta-carotene (mcg)..mean..SD.. |  | 2344.291 (4265.526) | 2030.820 (4580.021) | **0.038** |
| VCI (mg)..mean..SD. | Q1 | 2414 (24.400) | 286 (32.600) | **<0.001** |
|  | Q2 | 2446 (24.800) | 241 (27.500) |  |
|  | Q3 | 2490 (25.200) | 194 (22.100) |  |
|  | Q4 | 2531 (25.600) | 155 (17.700) |  |
| Vitamin K (mcg)..mean..SD.. |  | 124.583 (176.176) | 103.787 (134.309) | **0.001** |
| Calcium (mg).. |  | 898.833 (563.670) | 849.705 (583.464) | **0.014** |
| Potassium(mg)..mean..SD.. |  | 2522.758 (1267.217) | 2356.852 (1295.346) | **<0.001** |
| Coronary heart disease.... | No | 9504 (96.200) | 735 (83.900) | **<0.001** |
|  | Yes | 377 ( 3.800) | 141 (16.100) |  |
| Malignancy.... | No | 8720 (88.300) | 689 (78.700) | **<0.001** |
|  | Yes | 1161 (11.700) | 187 (21.300) |  |
| Smoke.... | No | 6116 (61.900) | 227 (25.900) | **<0.001** |
|  | Yes | 3765 (38.100) | 649 (74.100) |  |
| **Vitamin D (mg)..mean..SD..** |  | 4.44 (5.52) | 4.27 (5.96) | 0.390 |
| **Magnesium (mg)..mean..SD..** |  | 296.56 (155.13) | 263.88 (148.40) | **<0.001** |

VCI, Vitamin C Intake: Q1 (0 ≤ VCI < 19.3), Q2 (19.3 ≤ VCI < 50.1), Q3 (50.1 ≤ VCI < 110.6), Q4 (110.6 ≤ VCI ≤ 1977.4) (with added VitaminD and Magnesium).

Figure S2 A

**
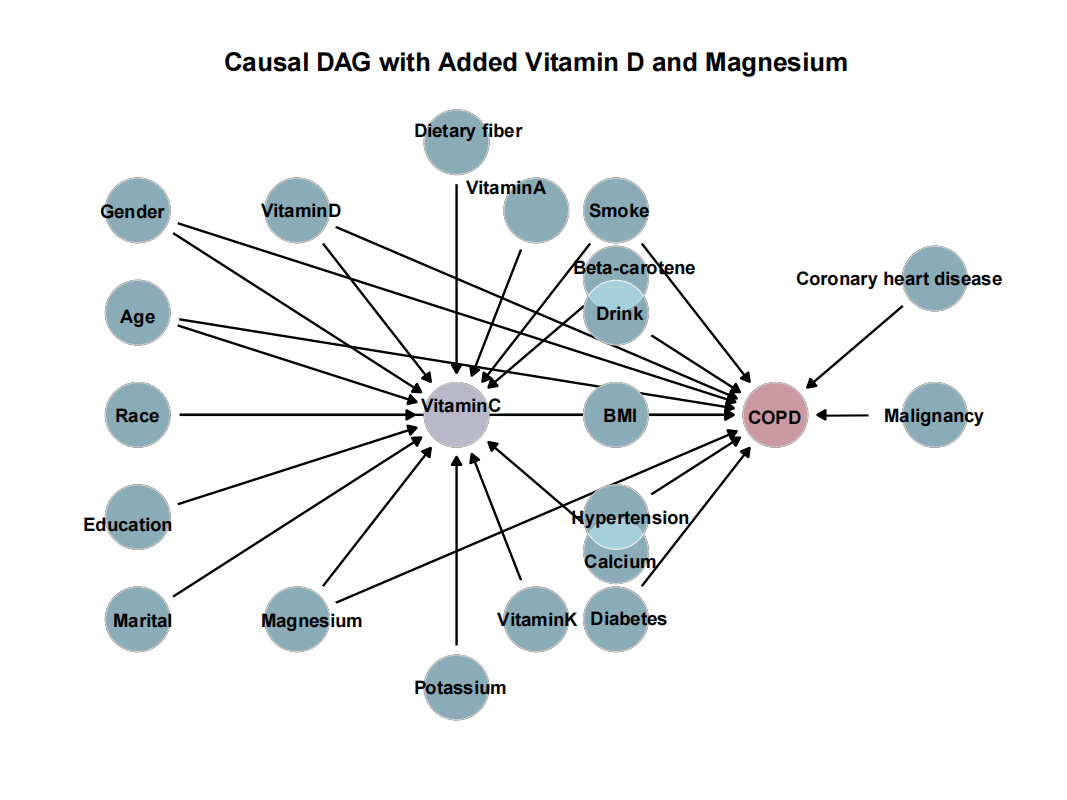
**

DAG for pre-identifying covariates (with added VitaminD and Magnesium).

Notes: It can be concluded from the graph that both vitamin D and magnesium are confounding variables and must be controlled. Therefore, we have added an analysis of vitamin D and magnesium as covariates in the supplementary document.

Figure S2 B


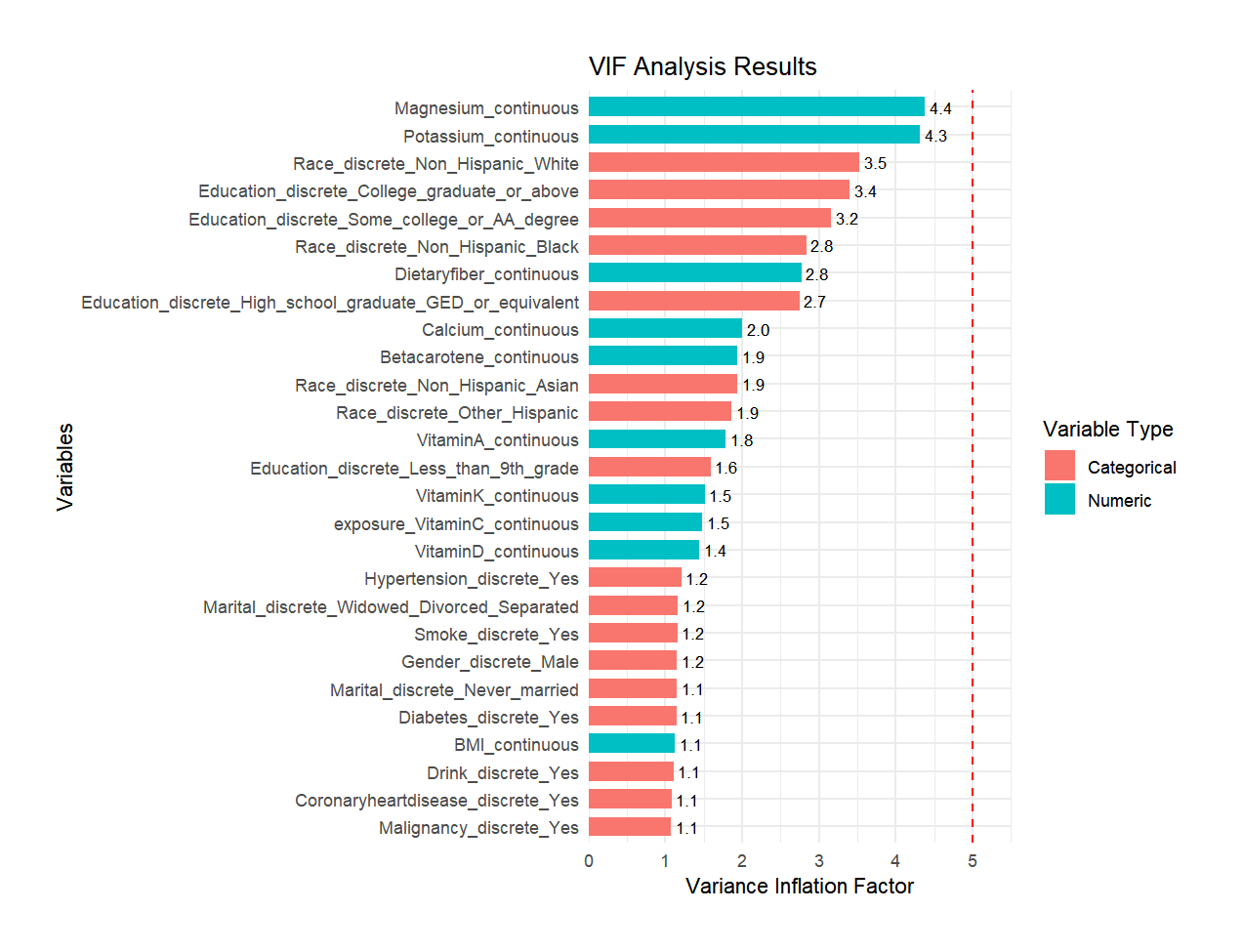


Conduct Variance Inflation Factor (VIF) detection for all variables (with added VitaminD and Magnesium).

Figure S2 C


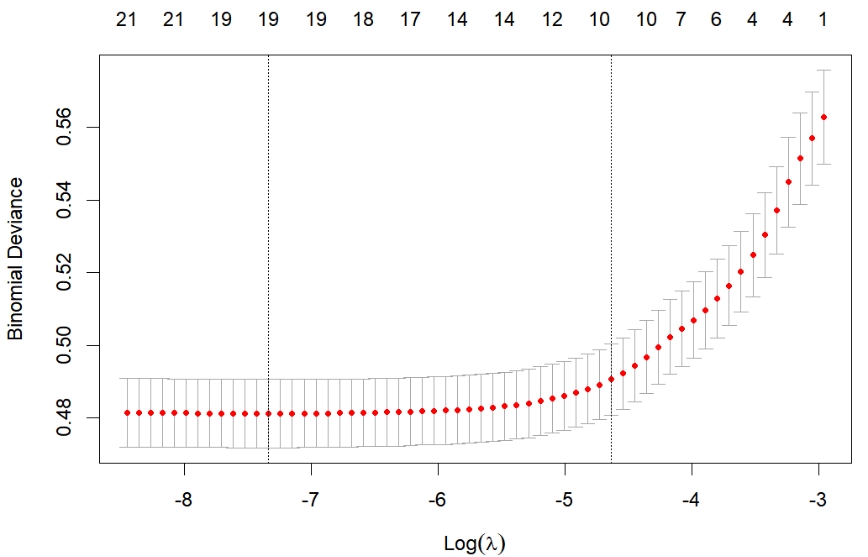


Lasso 10-fold cross-validation plot (with added VitaminD and Magnesium).

Figure S2 D


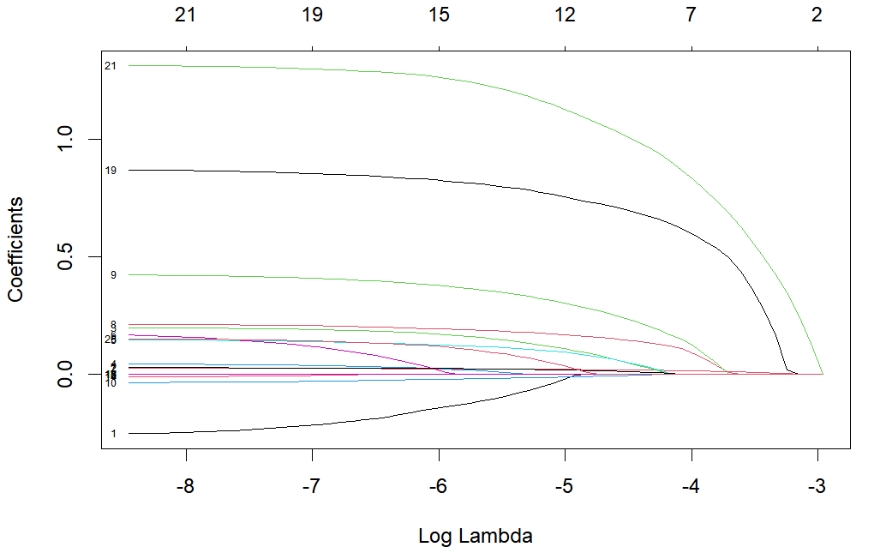


Lasso regression path plot (with added VitaminD and Magnesium).

Figure S2 E


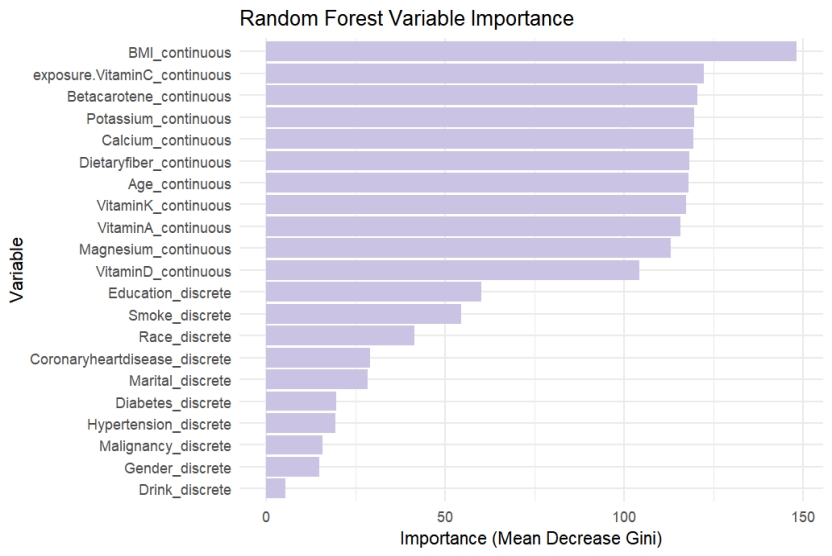


Random Forest variable importance plot (with added VitaminD and Magnesium).

Figure S2 F


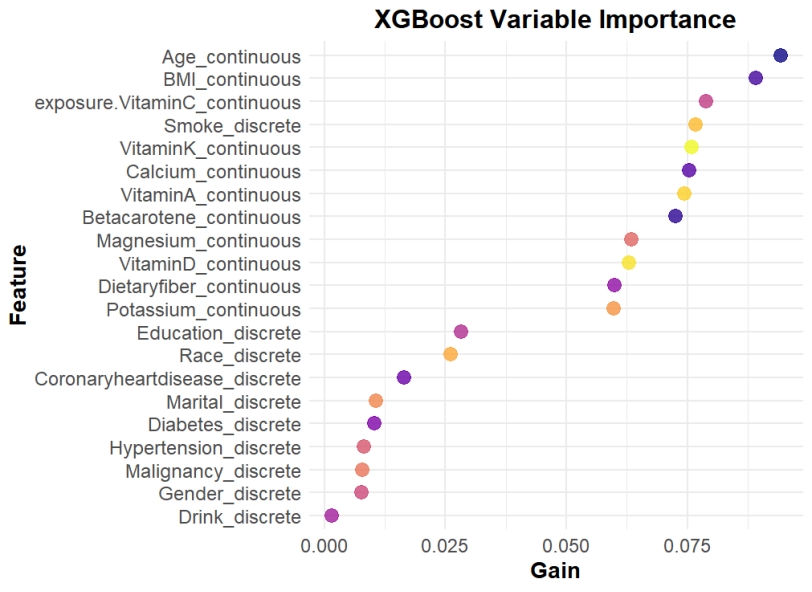


Variable importance plot for an XGBoost model (with added VitaminD and Magnesium).

Figure S2 G


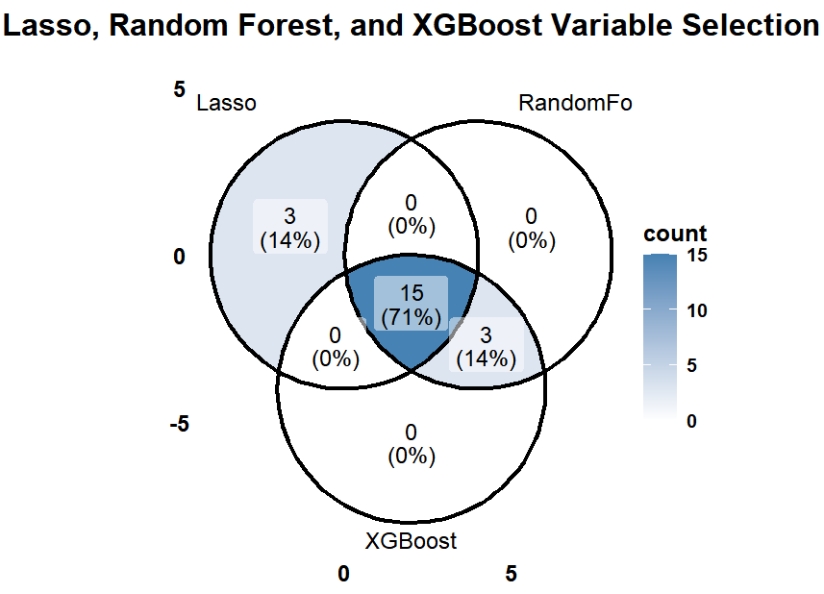


Three algorithmic Venn diagram screening variables (with added Vitamin D and Magnesium).

1. Lasso: Gende, Age, Race, Education, Marital, Drink, BMI, Hypertension, Diabetes, Dietaryfiber, VitaminA, exposure.VitaminC, **VitaminD**, VitaminK, Calcium, Potassium, Coronaryheartdisease, Malignancy, Smoke.
2. Random Forest: BMI, exposure.VitaminC, Betacarotene, Potassium, Calcium, Dietaryfiber, Age, VitaminK, VitaminA, **Magnesium, VitaminD**, Education, Smoke, Race, Coronaryheartdisease, Marital, Diabetes, Hypertension.
3. XGBoost: Age, BMI, exposure.VitaminC, Smoke, VitaminK, Calcium, VitaminA, Betacarotene, **Magnesium**, **VitaminD**, Dietaryfiber, Potassium, Education, Race, Coronaryheartdisease, Marital, Diabetes, Hypertension.
4. Venn: **Age, Race, Marital, BMI, Hypertension, Diabetes, Dietaryfiber, VitaminA, exposure.VitaminC, VitaminD, VitaminK, Calcium, Potassium, Coronaryheartdisease, Smoke. (Finally, we found through the variables screened by machine learning that magnesium was still not included. )**

Figure S3


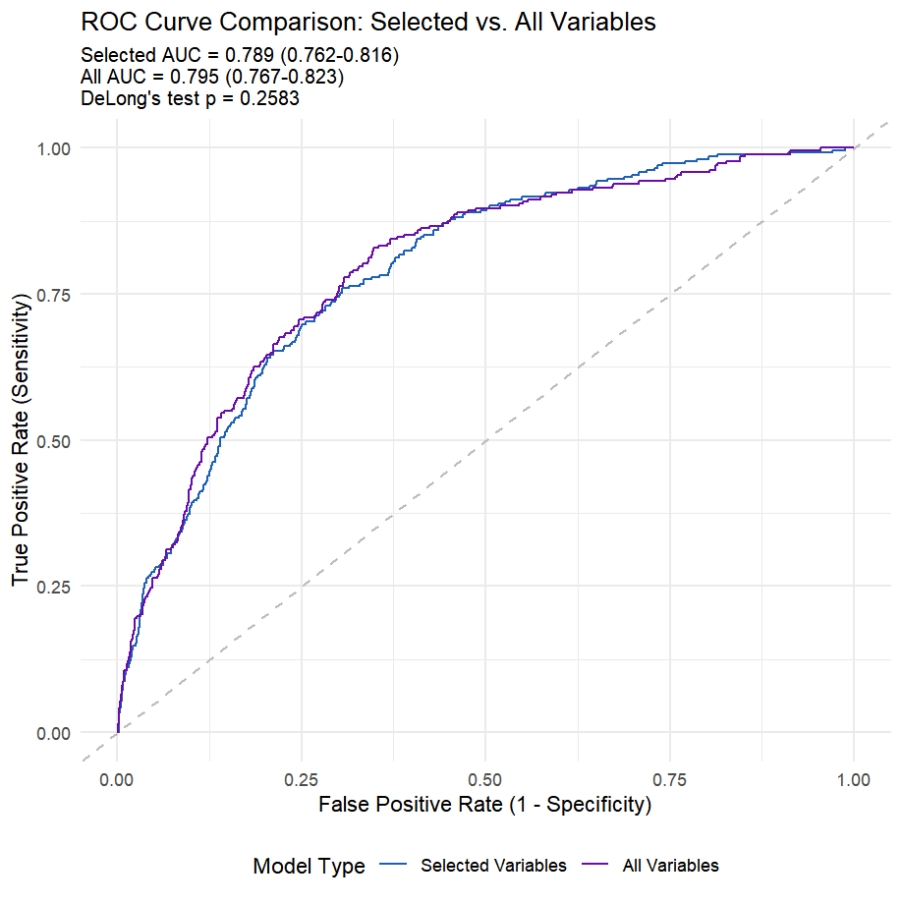
The Receiver Operating Characteristic (ROC) curves of machine learning (selected variables) and all variables models.

| Supplementary Table 4  Multivariate logistic regression analysis of the linkage betweenVCI and COPD. | | | | | | |
| --- | --- | --- | --- | --- | --- | --- |
|  | model1 | | model2 | | model3 | |
| Total VCI quantile | OR(95%*CI*) | P value | OR(95%*CI*) | P value | OR(95%*CI*) | P value |
| Q1 | ref |  | ref |  | ref |  |
| Q2 | 0.754(0.569-1.000) | 0.057 | 0.778(0.584-1.037) | 0.100 | 0.825(0.608-1.118) | 0.229 |
| Q3 | 0.640(0.503-0.814) | **0.001** | 0.711(0.551-0.918) | **0.016** | 0.707(0.519-0.963) | **0.041** |
| Q4 | 0.489(0.356-0.671) | **0.000** | 0.567(0.413-0.777) | **0.002** | 0.574(0.384-0.860) | **0.014** |

Abbreviations: *CI*, confidence interval; OR, odds ratio

Model 1, unadjusted.

Model 2, adjust for gender, age, race, education, marital, drink, BMI.

Model 3, adjust for age, race, marital, BMI, hypertension, diabetes, dietary fiber, vitamin A, vitamin D, vitamin K, calcium, potassium, coronary heart disease, smoke. (Variables screened by three machine learning methods)

Figure S4

**
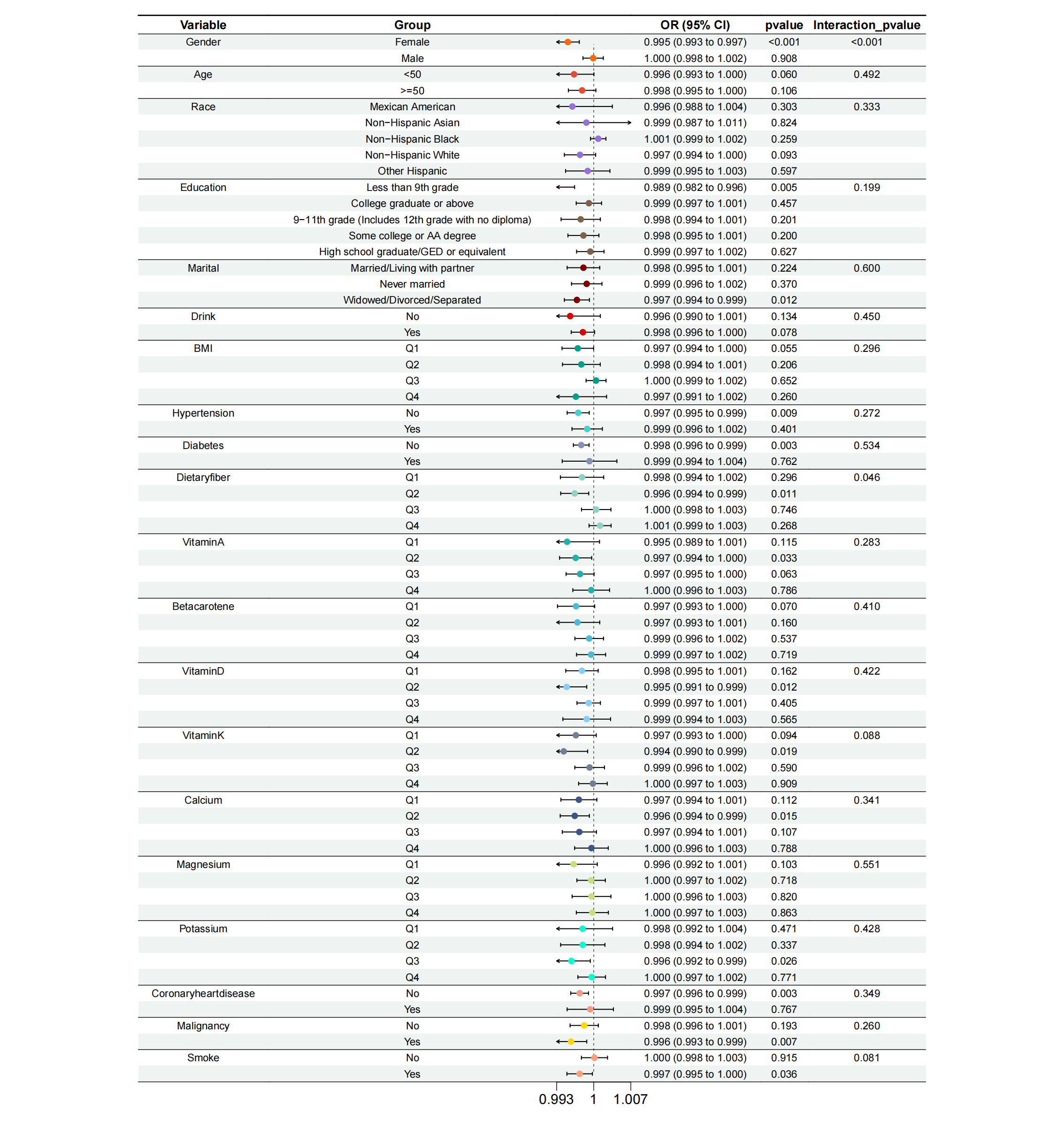
**

Subgroup analysis of the relationship between calcium intake, and COPD risk at different levels (with added Vitamin D and Magnesium).

Figure S5 A

**
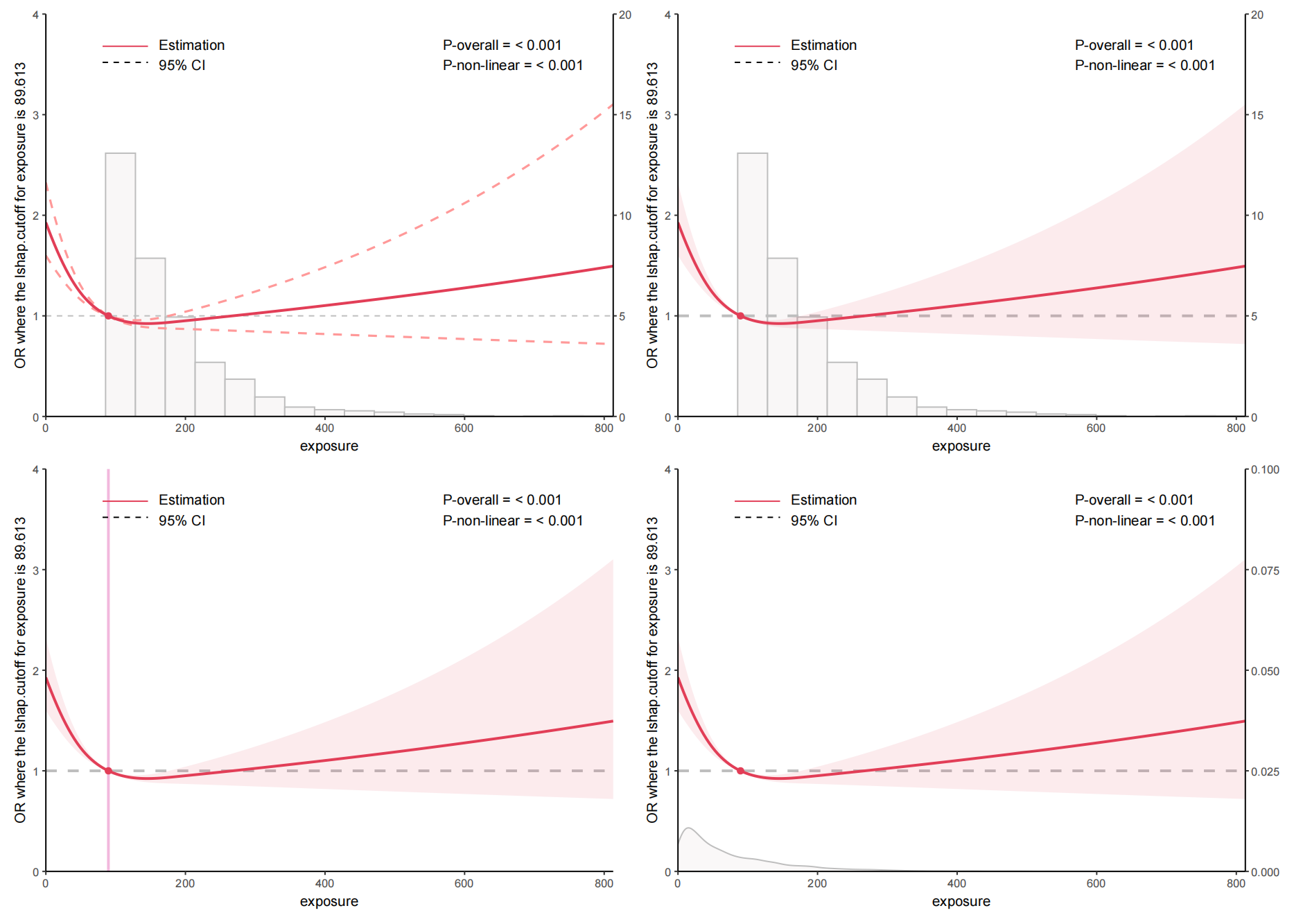
**

**
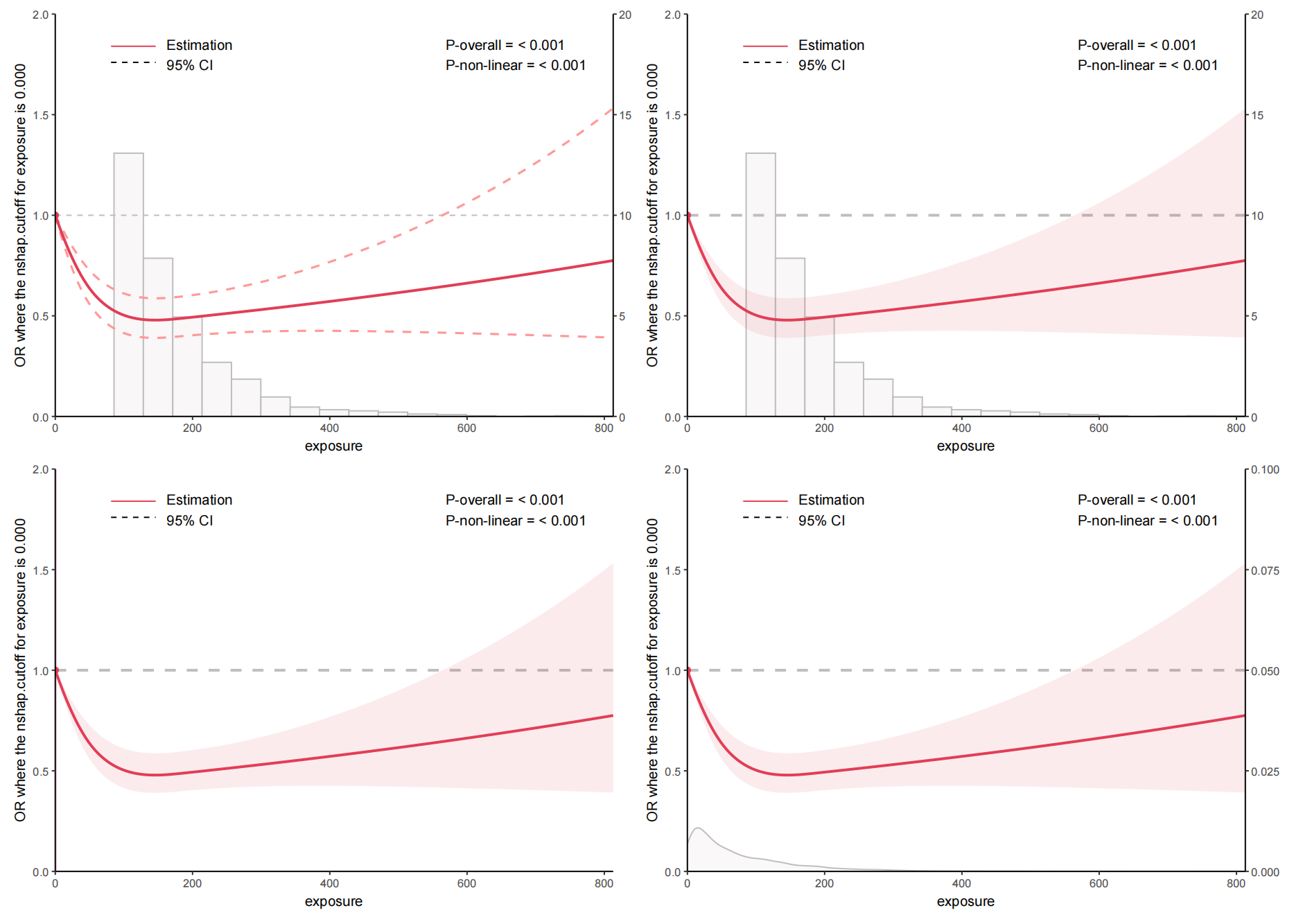
**

**
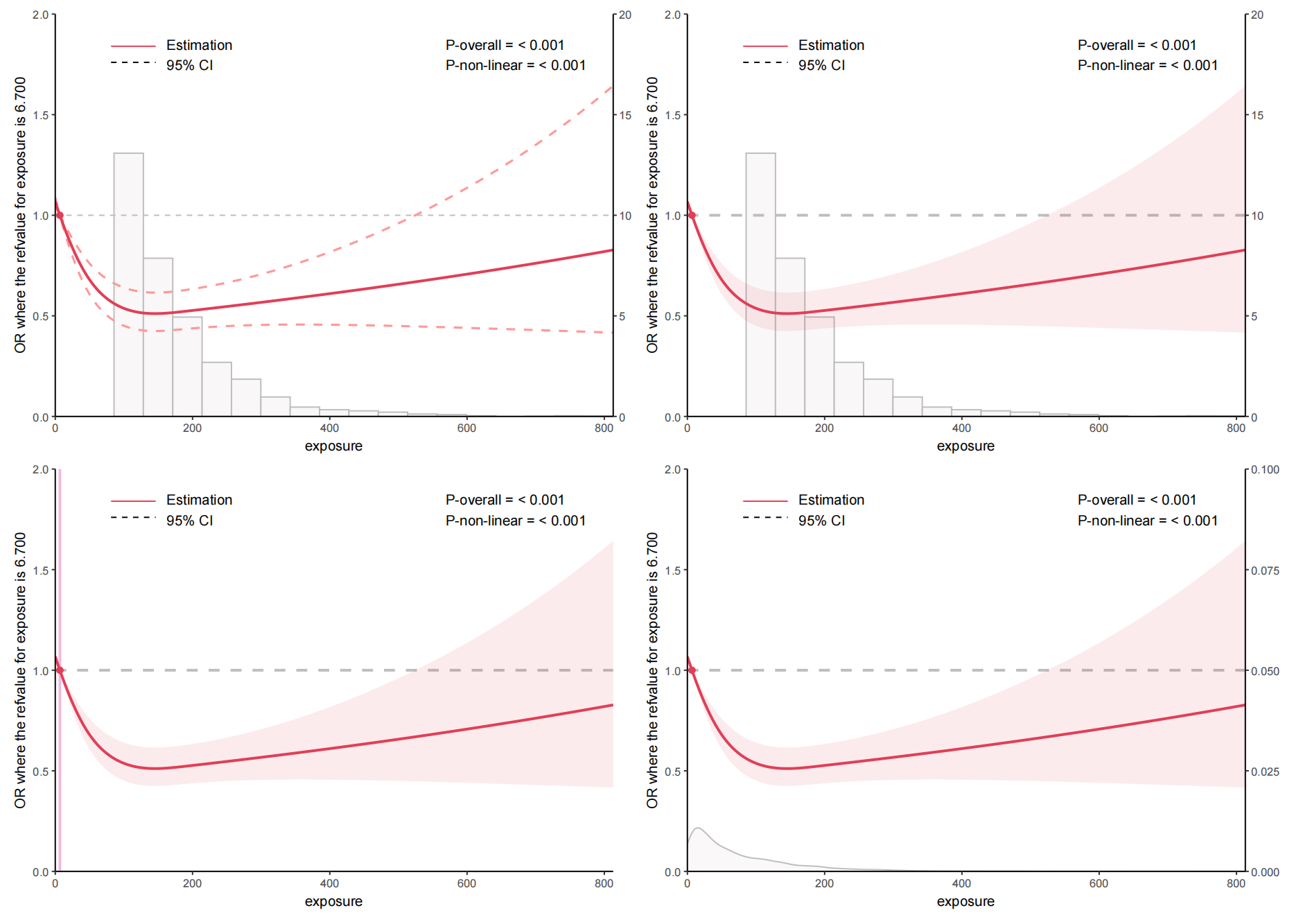
**

**
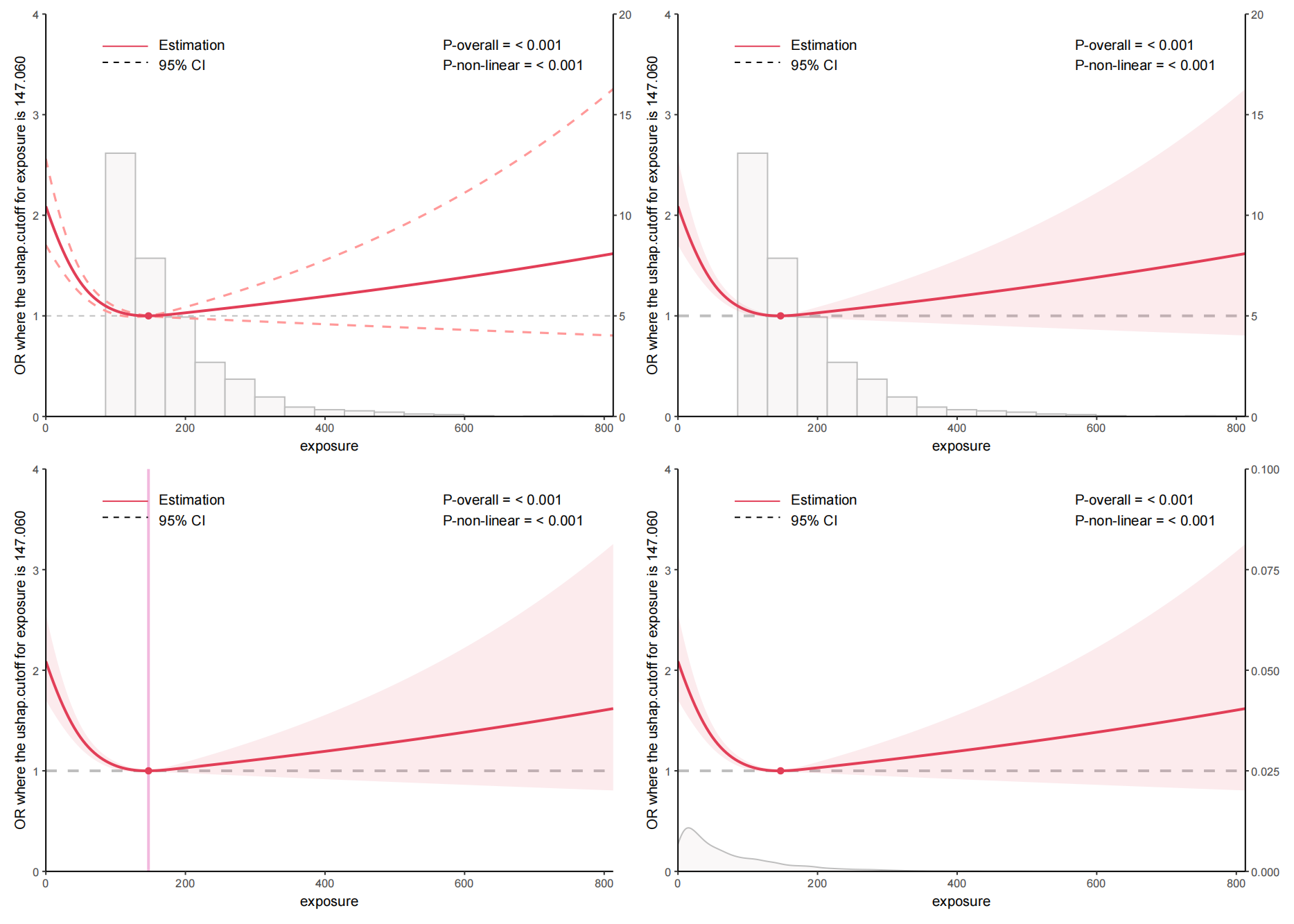
**The Restricted Cubic Splines (RCS) curve shows the association between VCI and COPD in all study participants. We did not conduct variable adjustment.

Figure S5 B

**
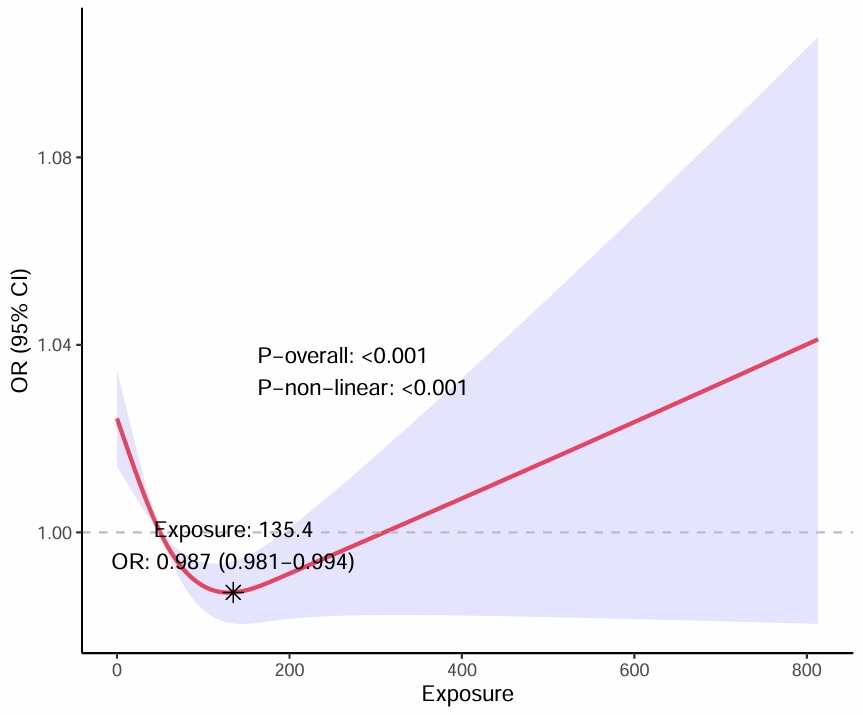
**

Adjust for age, race, marital, BMI, hypertension, diabetes, dietary fiber, vitamin A, vitamin D, vitamin K, calcium, potassium, coronary heart disease, smoke.

Supplementary Table 5

Threshold analysis result

| Outcome | effect | *P* |
| --- | --- | --- |
|  |  |  |
| Model 1 Fitting model by standard linear regression | 0.999 (0.998 - 1.000) | 0.268 |
| Model 2 Fitting model by two-piecewise linear regression |  |  |
| Inflection point | 135.400 |  |
| <135.400 | 0.996 (0.993 - 0.999) | **0.002** |
| ≥135.400 | 1.001 (0.999 - 1.002) | 0.396 |
| P for likelihood test |  | **<0.001** |

Notes: adjust for age, race, marital, BMI, hypertension, diabetes, dietary fiber, vitamin A, vitamin D, vitamin K, calcium, potassium, coronary heart disease, smoke.

③ The following is a summary of all the abbreviations in the original manuscript:

- **All abbreviations:**

Vitamin C Intake (VCI)

Chronic Obstructive Pulmonary Disease (COPD)

Restricted Cubic Spline (RCS)

Compound Annual Growth Rate (CAGR)

Recommended Dietary Allowance (RDA)

National Institutes of Health (NIH)

Centers for Disease Control and Prevention (CDC)

National Center for Health Statistics (NCHS)

Forced Expiratory Volume in one second (FEV1)

Forced Vital Capacity (FVC)

Directed Acyclic Graphs (DAGs)

Variance Inflation Factor (VIF)

Receiver Operating Characteristic (ROC)

Area Under the Receiver Operating Characteristic Curve (AUC)

Reactive Oxygen Species (ROS)
